# Outcome Prediction in Pneumonia Induced ALI/ARDS by Clinical Features and Peptide Patterns of BALF Determined by Mass Spectrometry

**DOI:** 10.1371/journal.pone.0025544

**Published:** 2011-10-03

**Authors:** Jochen Frenzel, Christian Gessner, Torsten Sandvoss, Stefan Hammerschmidt, Wolfgang Schellenberger, Ulrich Sack, Klaus Eschrich, Hubert Wirtz

**Affiliations:** 1 Institute of Biochemistry, Medical Faculty, University of Leipzig, Leipzig, Germany; 2 Department of Respiratory Medicine, University of Leipzig, Leipzig, Germany; 3 Institute of Clinical Immunology, University of Leipzig, Leipzig, Germany; University of Giessen Lung Center, Germany

## Abstract

**Background:**

Peptide patterns of bronchoalveolar lavage fluid (BALF) were assumed to reflect the complex pathology of acute lung injury (ALI)/acute respiratory distress syndrome (ARDS) better than clinical and inflammatory parameters and may be superior for outcome prediction.

**Methodology/Principal Findings:**

A training group of patients suffering from ALI/ARDS was compiled from equal numbers of survivors and nonsurvivors. Clinical history, ventilation parameters, Murray's lung injury severity score (Murray's LISS) and interleukins in BALF were gathered. In addition, samples of bronchoalveolar lavage fluid were analyzed by means of hydrophobic chromatography and MALDI-ToF mass spectrometry (MALDI-ToF MS).

Receiver operating characteristic (ROC) analysis for each clinical and cytokine parameter revealed interleukin-6>interleukin-8>diabetes mellitus>Murray's LISS as the best outcome predictors. Outcome predicted on the basis of BALF levels of interleukin-6 resulted in 79.4% accuracy, 82.7% sensitivity and 76.1% specificity (area under the ROC curve, AUC, 0.853). Both clinical parameters and cytokines as well as peptide patterns determined by MALDI-ToF MS were analyzed by classification and regression tree (CART) analysis and support vector machine (SVM) algorithms. CART analysis including Murray's LISS, interleukin-6 and interleukin-8 in combination was correct in 78.0%. MALDI-ToF MS of BALF peptides did not reveal a single identifiable biomarker for ARDS. However, classification of patients was successfully achieved based on the entire peptide pattern analyzed using SVM. This method resulted in 90% accuracy, 93.3% sensitivity and 86.7% specificity following a 10-fold cross validation (AUC = 0.953). Subsequent validation of the optimized SVM algorithm with a test group of patients with unknown prognosis yielded 87.5% accuracy, 83.3% sensitivity and 90.0% specificity.

**Conclusions/Significance:**

MALDI-ToF MS peptide patterns of BALF, evaluated by appropriate mathematical methods can be of value in predicting outcome in pneumonia induced ALI/ARDS.

## Introduction

Patients with acute lung injury (ALI) and acute respiratory distress syndrome (ARDS) are at increased risk of death within 28 days [1]–[3]. A systematic analysis of the ALI/ARDS literature, including 72 studies with more than 30 patients, yielded an overall pooled mortality rate of 43% [4], [5]. In patient series that do not include trauma patients pneumonia and sepsis are the major causes of ALI/ARDS and these patients appear to do worse than trauma patients [3]. Early indicators of prognosis may help to select appropriate treatment strategies. Indeed the treatment of ALI/ARDS is based on clinical severity and tailored to ventilatory parameters, key organ function and the evolution of oxygenation eventually requiring increasingly specialized management options [6].

Clinical predictors of adverse clinical outcome in ALI/ARDS are age greater 70, comorbidities including chronic liver disease and prior immunosuppression as well as the degree of multisystem organ failure. An elevated McCabe score for underlying disease, higher values of the acute physiology and chronic health evaluation score (APACHE III), the sequential organ failure assessment score (SOFA) and indications of fibroproliferative activity in the lung have been associated with poor outcome [1], [2], [7] (see [8] for a brief summary). Murray's lung injury severity score (Murray's LISS) which consists of three distinct clinical parameters still represents one accepted means of severity estimation [9]. Recently, predicted extravascular lung water (ELW) and the oxygenation index (OI) have also been shown to be independent predictors of mortality in ALI [2], [10], [11]. Several biological markers have been described and among those were markers of inflammation (IL-6, IL-8, IL-10, TNF-α, soluble TNF receptors I and II), lung epithelial injury (surfactant protein D, receptor for advanced glycation end products, RAGE), adhesion molecules (intercellular adhesion molecule 1, ICAM-1), activation of coagulation and inhibition of fibrinolysis (protein C, plasminogen activator inhibitor-1, PAI-1), von Willebrand factor, procollagen peptide III (P3NP) and brain natriuretic peptide (BNP) [7], [12]–[14] (see [2] for review).

Diagnostic information may also be obtained from analysis of the bronchoalveolar lavage fluid (BALF) recovered during bronchoscopy indicated e.g. for microbiological reasons. BALF represents greatly diluted airway/alveolar epithelial lining fluid and contains cells, lipids, nucleic acids and peptides/proteins. BALF most faithfully reflects the peptide/protein composition in the airways and alveoli [15], [16]. These peptides arise either directly from the airways/alveoli or from serum via translocation. Some of these molecules may be markers of lung disease. Recently, flow cytometry, gene expression arrays, and proteomics were all applied to BALF, pulmonary edema fluid and serum in order to identify peptides or proteins which are up- or downregulated significantly in various pulmonary diseases [17], [18]. Proteomic approaches to lung diseases typically have applied two-dimensional electrophoresis for protein separation followed by identification of differentially expressed proteins by mass spectrometry (MS) [19]. Healthy individuals [15], [18]–[21] and patients with asthma [22], chronic obstructive pulmonary disease (COPD) [23], [24], cystic fibrosis, sarcoidosis [25] and sepsis/ARDS [18], [19], [26]–[28] were investigated. In some studies MS spectra of complex samples from lung patients were recorded and the peptide/protein patterns were analyzed by means of mathematical algorithms without peak identification. These techniques are termed ‘peptide/protein profiling’ [15]. Profiling of plasma proteins successfully distinguished patients with COPD [29] and lung cancer [30] from healthy volunteers. Protein patterns from 15 distinct MS peaks were recognized to identify different types of non-small-cell lung cancer and groups with differing prognosis [31], [32]. Differences in the relative abundance of proteins in survivors versus nonsurvivors in ALI/ARDS were expected to be subtle.and peptide profiling via MS was thought to be a method particularly suitable for that purpose.

We started with predicting outcome of pneumonia induced ALI/ARDS patients on the basis of clinical data including Murray's LIS score, ventilation parameters, i.e. peak inspiratory pressure (PIP), positive end-expiratory airway pressure (PEEP), risk factors (diabetes mellitus, smoking habit) and BALF interleukins. A new approach was then developed to predict outcome of patients from BALF peptide patterns captured by mass spectrometric determination and analyzed by sophisticated mathematical methods. This peptide profiling approach is fast and proved superior following cross validation, calculation of receiver operating characteristic (ROC) curves and the area under the ROC curve (AUC) and validation by analyzing a test group of patients [7], [33]–[35].

## Results

### Outcome Prediction Based on Clinical Features

**Figure 1:**
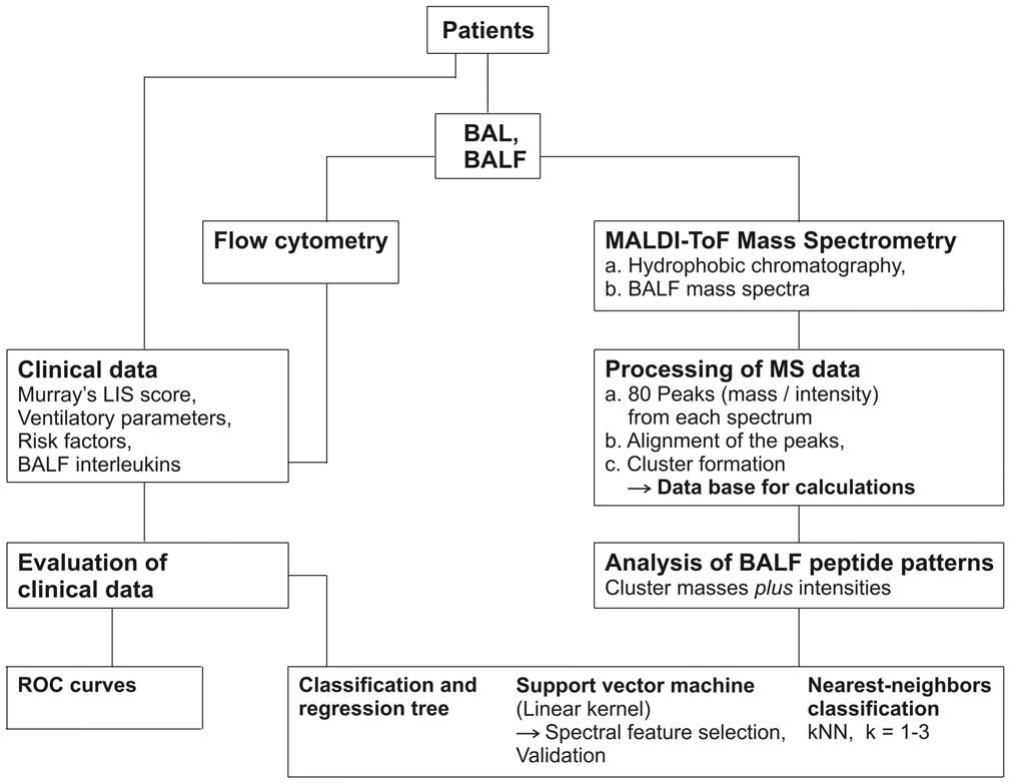
Data mining from the patients. Processing of generally clinical data and interleukins and peptide pattern from BALF. The diagram illustrates sample preparation, data processing and analysis by statistics and different mathematical algorithms. Best results were obtained by applying a support vector machine (SVM) to peptide patterns. The SVM classifier was optimized with the patterns from the patients of the training group. The performance of the classifier was then validated by patterns of the patients from a subsequently recruited test group. BAL, bronchoalveolar lavage; BALF, bronchoalveolar lavage fluid; ROC, receiver operating characteristic curve.

Individual risk factors, Murray's LIS score, ventilatory variables and inflammatory parameters in BALF were gathered from 30 patients with pneumonia induced ALI/ARDS. This training group was compiled from 15 survivors and 15 nonsurvivors on the basis of 28 day outcome. The design of the study is shown in Figure 1.

**Figure 2:**
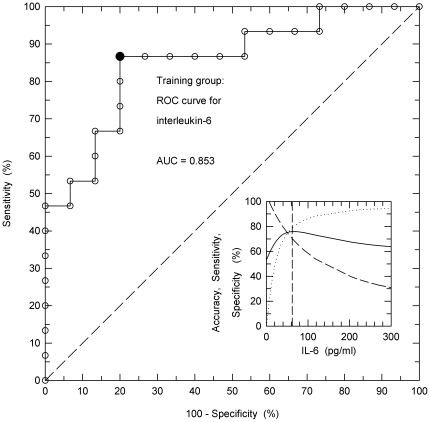
Receiver operating characteristic curve for interleukin-6. The receiver operating characteristic (ROC) curve for IL-6 as an important clinical parameter of outcome prognosis was computed from the raw data of the 30 patients of the training group. The closed symbol indicates the point at IL-6, 62.4 pg/ml which classifies best. Insert: ROC curves for accuracy (solid line), sensitivity (dashed line) and specificity (dotted line) in dependence on the IL-6 concentration in the BALF. The vertical dashed line indicates best separation at optimum discrimination value of IL-6 with an accuracy of 83.3%. Accuracy defines the percentage of true positives and true negatives related to all patients.

To identify powerful binary classifiers for outcome receiver operating characteristic (ROC) analysis was performed for each clinical and cytokine parameter. (Table 1). Interleukin-6 (IL-6) was found to be the best single parameter for outcome prediction. Figure 2 demonstrates accuracy, sensitivity and specificity over the entire range of IL-6 concentrations. The vertical dashed line defines the optimum predictive value of all these qualities with 82.7% sensitivity, 76.1% specificity and 79.4% accuracy. The area under the ROC curve (AUC) was 0.853. At the onset of ARDS levels of IL-6 were significantly increased in the 15 nonsurvivors (median: 246, range: 8–1250 pg/ml) compared with those in the 15 survivors (median: 20, range: 6–56 pg/ml). Table 1 depicts BALF and clinical parameters and the statistical significance between survivors and nonsurvivors of each of these parameters. While IL-6, IL-8, Murray's LISS, peak inspiratory pressure (PIP), positive end-expiratory airway pressure (PEEP), the presence/absence of a concomitant diabetes mellitus and smoking history were significantly different between nonsurvivors and survivors, age, BMI, IL-1β, IL-10, IL-12 and TNF-α were not different among the two groups.

**Table 1:** Statistical analysis of clinical parameters and cytokines of the patients of the training group.

*Parameter*	*Measure*	*Correct rate (%)* 1	*AUC*	*Parameter value* 2	*Mean*	*Std.dev*	*Mean*	*Std.dev*	*Significance* 3
					*Survivors*		*Nonsurvivors*		
IL-6	pg/ml	79.4	0.853	62.4	95	223	782	1202	*
IL-8	pg/ml	76.7	0.813	1290.2	780	867	3213	2704	*
Age	years	76.7	0.633	72.1	62.1	7.1	64.4	21.0	
Diabetes mell.		73.3		0	0.07	0.26	0.53	0.52	*
Murray's LISS		70.0	0.733	2.7	2.0	0.9	2.7	0.7	*
PIP	mbar	70.0	0.753	23.2	20.2	3.9	25.0	5.6	*
Smoking		70.0		0	0.40	0.51	0.80	0.41	*
PEEP	mbar	66.7	0.742	8.0	9.0	2.7	12.4	5.3	*
IL-1β	pg/ml	66.7	0.800	42.5	33	48	332	1020	
IL-12	pg/ml	66.7	0.696	4.3	3.5	2.8	5.5	5.3	
TNF-α	pg/ml	66.7	0.616	2.8	7.4	12.2	12.1	28.1	
BMI	kg/m^2^	63.3	0.504	27.9	24.98	2.45	25.69	5.10	
IL-10	pg/ml	63.3	0.427	2.4	3.0	4.0	2.9	2.0	
Tidal volume	ml/kg BW	36.7	0.509	4.5	5.72	0.69	5.62	1.38	

PIP indicates peak inspiratory airway pressure, BMI indicates body mass index.

1 accuracy without cross validation at optimum discrimination value.

2 optimum discrimination value.

3 comparison of nonsurvivors versus survivors: *P*<0.05.

A classification tree including IL-6, IL-8 and Murray's LISS yielded an accuracy of 93,3%. The calculated ROC curve is shown in Figure S1. To refine the performance estimation and to consider the risk of overfitting cross validation was applied. Following a 10-fold cross validation, accuracy decreased to 79.3%. Alternatively, a random forest classification or a support vector machine (SVM) algorithm using the clinical features both led to 81% accuracy. In order to facilitate the comparison of our results with those of other authors we also calculated the areas under the receiver operating characteristic (ROC) curves (AUC). AUC may represent the most popular measure for the performance of binary classifiers [7], [33]–[35]. AUC values of at least 0.8–0.85 without cross validation identify predictors with high prognostic potential [7], [14].

### Outcome Prediction by Means of the Mass Spectrometric Approach

Mass spectra were acquired from BALF of the training group detailed above. Concentrated BALF was used both with and without subsequent purification using hydrophobic interaction chromatography. Mass spectra obtained from raw and purified BALF were essentially similar, but the latter showed additional peaks between *m/z* 5,500 and 8,000 Da and overall improved signal to noise ratio as well as peak width in half-height. The spectra from purified BALF were clearly better for subsequent evaluation.

**Figure 3:**
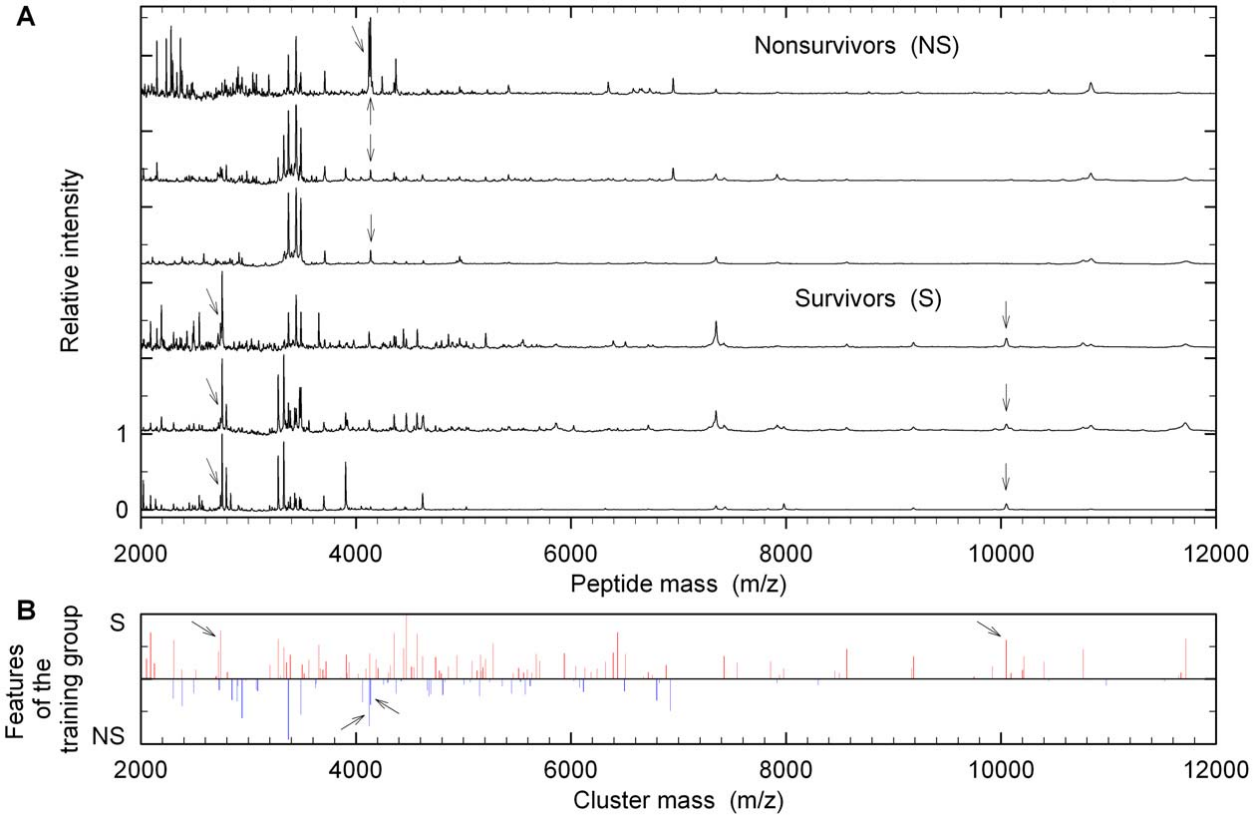
Presentation of typical mass spectra of BALF from patients of the training group. (A) Three examples of nonsurvivors and three examples of survivors are depicted. Peaks indicated by arrows at *m/z*, 2740.0 and 10049.9 are the most typical spectral features (cluster masses *plus* intensities) for survivors while *m/z*, 4121.6/4135.6 are most typical spectral features for nonsurvivors. (B) All spectral features in the mass spectra of the training group calculated by the clustering procedure and the spider algorithm. The lines running downwards (blue) are representative for the class nonsurvivors (NS), whereas the lines running upwards (red) are characteristic for the class survivors (S).

Figure 3A shows three examples of spectra from nonsurvivors (upper part) and from survivors (lower part). Numbers represent masses of peptides in BALF. Individual BALF spectra differed remarkably from each other, even within the survivor or nonsurvivor groups. Classification by mere visual inspection of spectra therefore was found to be not possible. Instead, spectra were analyzed and grouped by various mathematical algorithms (see below). First peak lists of all spectra were generated and peaks were assigned to clusters as described in “Materials and Methods”.

### Mathematical Analysis of Peptide Patterns in BALF

**Figure 4:**
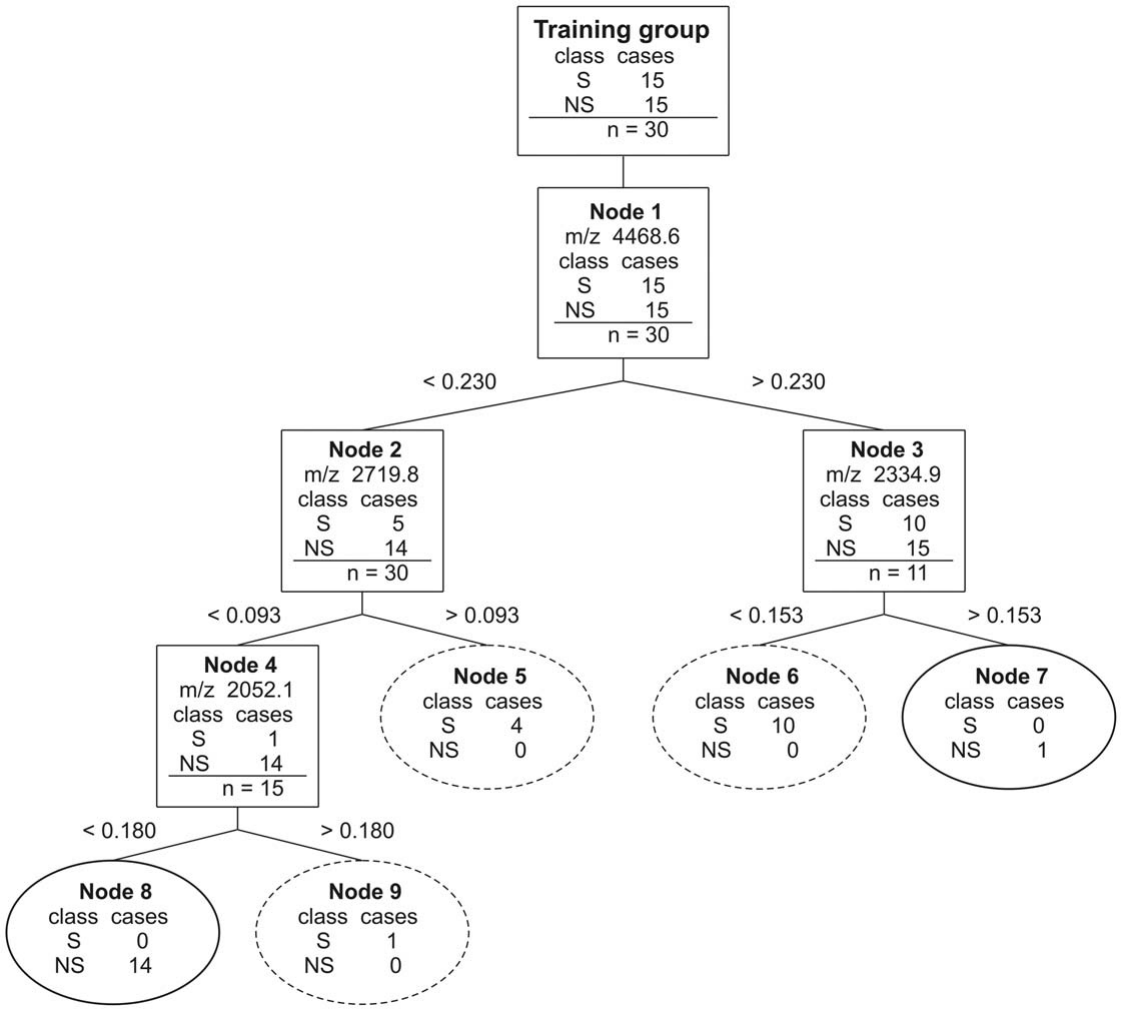
Best classification tree for the training group using MALDI-ToF MS data from BALF. Four cluster masses (mass peaks) were used to construct the tree (*m/z*, 4468.6, 2719.8, 2052.1 and 2334.9). The nodes were sequentially labelled on the basis of the branching level and show splitting criteria. As an example, *m/z* 4468.6<0.230 means that BALF with peak intensities lower than 0.230 at *m/z*, 4468.6 are allocated to the left branch and all other BALF to the right branch. BALF continue down the tree until they reach a terminal node depicted as ellipses. Ellipses with full lines denote terminal nodes of nonsurvivors. The number of BALF at each node are given for both survivors (S) and nonsurvivors (NS). The tree classifies all patients correctly, however, accuracy decreased to 76.7% after 10-fold cross validation.

**Figure 5:**
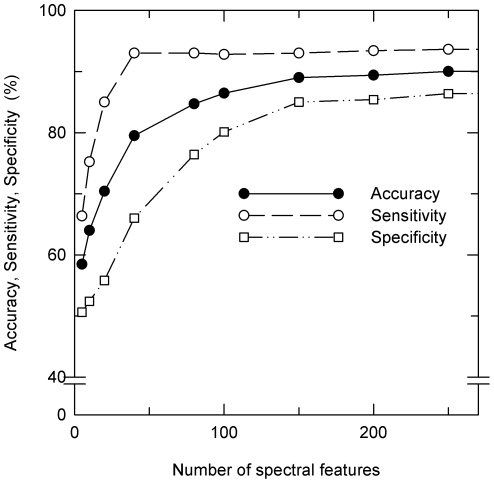
Effect of the spectral features on the accuracy of prognosis. Dependence of accuracy, sensitivity and specificity on the number of spectral features used by the SVM algorithm for classification of the individual patients of the training group on the basis of the BALF mass spectra. Accuracy, sensitivity and specificity have been obtained after 10-fold cross validation.

No exclusive cluster masses were identified in the nonsurvivor as well as the survivor groups. Figure 4 shows a classification tree obtained by classification and regression tree (CART) analysis. It was constructed from four cluster masses and led to zero misclassifications. However, accuracy decreased to 76.7% following 10-fold cross validation. Application of a nearest-neighbors classifier resulted in 73.3% correctness (Table 2). Finally, a support vector machine was trained with all cluster masses together with their intensities. It revealed spectral features (selected cluster masses *plus* intensities) suited for classification (Figure 3B). SVM proved to be superior to CART analysis. Following 10-fold cross validation, 90% accuracy, 93% sensitivity and 87% specificity (AUC, 0.953, Tables 2 and 3 and Figure 5) were achieved. Also for the classification tree shown in Figure 4 and for classification by SVM ROC curves were calculated which are presented in Figure S1. More data are summarized in Table S1.

**Table 2:** Classifications of the training group based on analysis of peptide pattern of BALF mass spectra with different mathematical methods.

*Classification algorithm*	*Accuracy* 1 *(%)*	*Sensitivity* 1 *(%)*	*Specificity* 1 *(%)*
CART analysis	70.0	67.0	67.0
Nearest-neighbors classifier (kNN; k = 3)	73.3	73.3	73.3
Support vector machine (linear kernel)	90.0	93.3	86.7

1 following 10-fold cross validation.

*P*<0.05.

**Table 3:** Measures of outcome prediction in ALI/ARDS and other lung diseases.

*Lung disease*					
*Predictors*	*Selected predictors*	*Algorithm*	*Accuracy (%)*	*AUC*	*Reference*
***ALI/ARDS***					
Clin. data, BALF cytokines	IL-6 (Training group)	ROC	79.4^1^	0.853^1^	This study
	IL 6 (Test group)		75.0^1^	n.a.	
	IL-6, IL-8, Murray's LISS	CART	89.0^2^	0.913^2^	
	(Training group)	CART	79.3	0.873	
	(Training group)	SVM	81.0	0.840	
BALF peptide patterns	Spectral features,	CART	100^2^	0.991^2^	
(MALDI-ToF MS)	(Training group)	CART	70.0	0.864	
	(Training group)	SVM	90.0	0.953	
	(Test group)		87.5	n.a.	
Clin. Data	Organ failure, age, …	ROC	–	0.815	Ware [7]
Plasma biol. markers	SP-D, IL6, IL-8, …		–	0.756	
	Clin. data/Biol. markers		–	0.850	
	Reduced model		–	0.811	
Clin. data	Predicted EVLW	ROC	–	0.800^1^	Craig [10]
Plasma biol. markers	RAGE receptor, IL-8, …	ROC	–	0.860^1^	Fremont [14]
BALF cytokines	IL-6	LRA	78.5^1^	0.730^1^	Lin [37]
	IL-8		75.5^1^	0.790^1^	
	Murray's LISS		79.0^1^	0.700^1^	
***Asthma, Obstructive lung disease, COPD***					
19 Predictors	Ever/current asthma,	CART	87.5^1^	–	Grassi [42]
	shortness of breath, …	Neuro. net	92.5^1^	–	
HRCT	Severe/mild centrilobular	Bayesian	81.2^5C^	–	Lee [44]
	emphysema, bronchiolitis obliterans, normal lung	SVM	83.1^5C^	–	
Plasma protein profiles	*m/z* and intensity	CART	90.0^1^	0.932^1^	
(SELDI-ToF MS)			81.7	–	Bowler [29]
***Lung cancer, NSCLC***					
Blood serum proteins (MALDI-ToF MS)	*m/z* and intensity	CART ROC	90.0	0.800	Markey [30]
Pept. profiling of lung	Top peaks,	CART			Gamez-Pozo
tissue (MALDI MS)	*m/z* and intensity	plus			[35]
	(All three diseases)	AdaBoost	93.9^LC^	–	
	(AC)		–	0.982^LC^	
	(LC)		–	0.991^LC^	
	(SC)		–	1.000^LC^	
Lung cancer tissue,	*CD34MVD*, *TIMP-2*,				Zhu [47]
	cytokeratins,…				
	(All patients)	SVM	87.2		
	(Validation cohort)		76.0		

HRCT, texture analysis at high-resolution computerized tomography; LRA, logistic regression analysis. AC, LC, SC, adenocarcinoma, large and squamous cell carcinoma; n.a., not applicable.

Accuracies and AUC; values include 10-fold>^5C^ 5-fold>^LC^ leave-one-out cross validation.

1 without cross validation (Simple ROC analysis comprises no cross validation.),

2 Cross validation was used to find out the optimal number of nodes.

The SVM algorithm also provided a ranking order of the spectral features (Figure 3B). The peaks at *m/z*, 9167.6, 4468.6, 6433.8, 2304.8, 1830.0, 4515.9, 2740.5, 4355.1, 10048.4 were more likely to be present in survivors while *m/z*, 5576.1, 4122.4, 2940.6, 2901.8, 6924.1, 4255.8, 3371.8, 4135.6, 4515.9 were preferentially found in nonsurvivors. However, these few peaks are not sufficient for an accurate outcome prediction in the ALI/ARDS training cohort (73.0% accuracy, compare Figure 5).

### Validation of the SVM Algorithm

**Figure 6:**
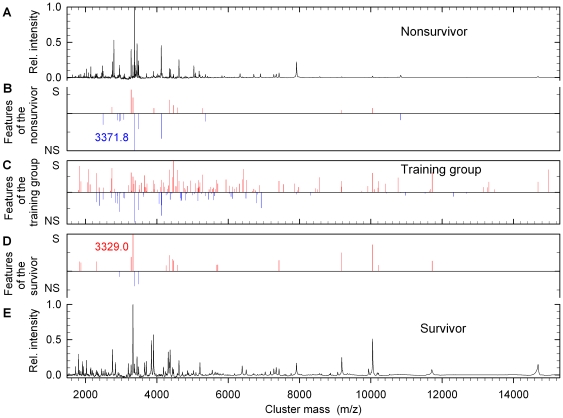
BALF mass spectra from two patients and calculated spectral features for outcome prediction. (A, E) Mass spectra of the BALF from a nonsurvivor and from a survivor. (B, D) The 20 most important spectral features found in (A) and (E). The lines running downwards (blue) are representative for the class nonsurvivors (NS), whereas the lines running upwards (red) are characteristic for the class survivors (S). (C) For comparison, all spectral features in the mass spectra of the training group calculated by the clustering procedure and the spider algorithm. Downward lines (blue) are representative for nonsurvivors (NS), upward lines (red) for survivors (S). The SVM algorithm considers both the occurrence and the absence of a spectral feature.

The performance of the SVM classifier was evaluated by applying it to a test group of 16 additional patients with unknown outcome. All samples were prepared in a single batch. 14 samples of the test group were correctly predicted. One survivor and one nonsurvivor were misclassified. For illustration, typical spectra from a survivor and a nonsurvivor are shown in Figure 6, parts A and E. In parts B and D the 20 most important spectral features identified by the SVM algorithm are depicted. The lines running downwards (blue) represent characteristic features of the nonsurvivor class, whereas the lines running upwards (red) indicate features of the survivor class. For comparison, part C comprises all spectral features derived from the training group. It should be noted that for classification of a sample the SVM algorithm considers not only the occurrence but also the absence of a feature.

**Figure 7:**
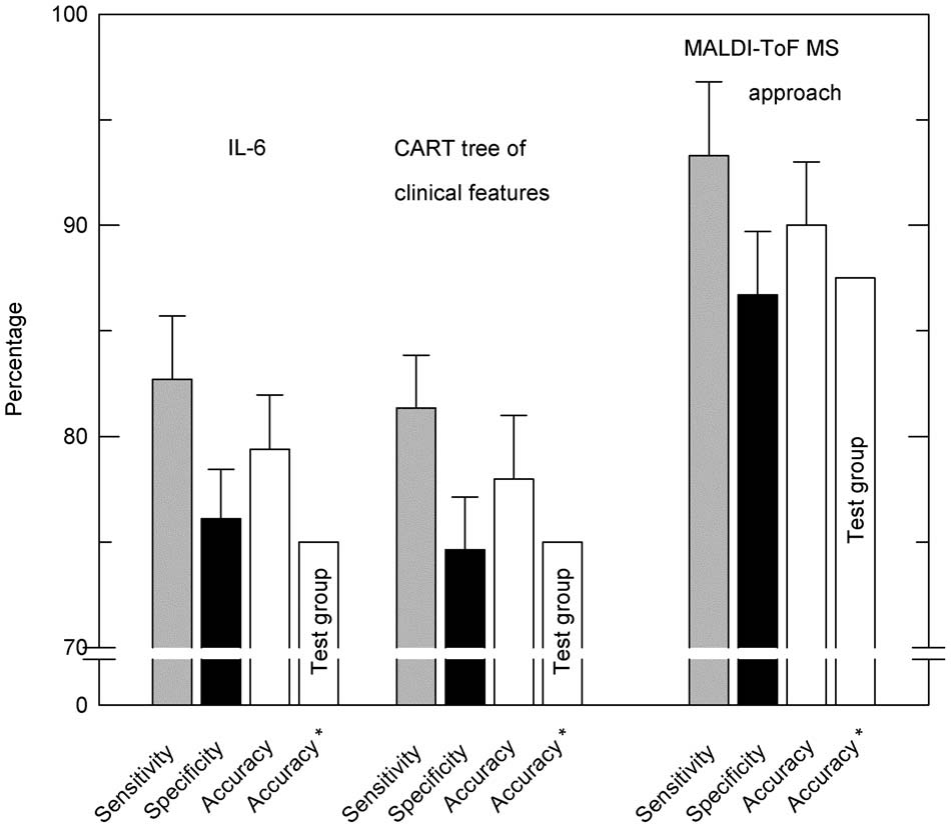
Prognosis by MALDI-ToF MS approach and clinical features for the training group. Accuracy, sensitivity and specificity of outcome prediction by pattern analysis of MALDI-ToF mass spectra of BALF in comparison to the results obtained on the basis of the interleukin-6 concentration and a classification tree of clinical features (IL-6, IL-8 and Murray's LISS). In addition, the accuracy (*) of outcome prediction of the test group is given. The error bars indicate SD after 10-fold cross validation.

As in the training group, outcome prediction of the test group based on clinical features was less precise than that achieved by the approach with matrix-assisted laser-desorption ionization time of flight mass spectrometry (MALDI-ToF MS, Figure 7).

## Discussion

Despite a decreasing mortality in recent years [4], ALI/ARDS is still a deadly disease and the early knowledge of a patient's prognosis from readily available clinical or laboratory data may be helpful in considering other options often termed “rescue therapies” or “unproven therapies” in ALI/ARDS [2], [6]. So far these therapies are employed as judged by the treating physician if standard therapies have failed. Prone position, high-frequency oscillatory ventilation, inhaled nitric oxide, extra corporal lung support and others tend to be used late in the process of ARDS and lack objective criteria for their use [6]. Although clinical features may distinguish some groups of patients with respect to age, body weight, ARDS pathophysiology or even lung injury score etc., this obviously has not led to criteria favouring one “rescue therapy” over another. A lung injury score ≥3 has been used to recommend the consideration of “rescue therapies”. More powerful and physician independent indices such as the mathematical evaluation of a complex pattern suggested in this study may provide better means for an early decision of the escalation pathway in patients with a poor prognosis and at the same time spare adverse effects in the rest. In addition, the classification of patterns of patients with retrospective benefit of a certain type of “rescue therapy” may in the future allow to predict which “rescue therapy” may have the greatest impact on survival.

Numerous studies of outcome predictors in ALI/ARDS in the past have involved one or more of the following: (i) calculations of the predictive power of single clinical risk factors alone or (ii) of single biological markers alone, (iii) combinations of clinical risk factors and biological markers, (iv) ‘peptide/protein profiling’ without peak identification, elements of proteomics, focused on estimating the severity of the disease and (v) complete proteomic approaches. In general, clinical risk factors, severity of illness scoring and diagnosis of sepsis have a moderate predictive value for death [7]. Predicted extravascular lung water (EVLW) was found to be a decent predictor for mortality in ALI [10]. The discriminatory power described by AUC was 0.8.

In our study ROC analysis of clinical parameters and cytokines revealed the Murray's LIS score as a good single clinical outcome predictor in ALI/ARDS. Murray's LISS has been recognized to be a useful indicator for morbidity in acute lung injury and following blunt thoracic trauma [36]. ROC analysis revealed a critical Murray's LISS of 2.5–2.7 (AUC = 0.733) as a predictor of fatal outcome similar to published values of 2.38 also analyzed by ROC [37] and of 2.76 obtained by logistic regression analysis [38].

The use of biological markers greatly improved the correctness of the outcome prognosis in ARDS. We found IL-6 and IL-8 in BALF to be the best outcome predictors by ROC analysis in ALI/ARDS (see Table 1). Similar findings have been reported in previous studies both in BALF [12], [37] and in plasma [7], [39] with regard to the interleukins. One advantage of plasma markers is that they are routinely determined. Seven plasma biomarkers (RAGE, P3NP, BNP, Ang-2, IL-10, TNF-α, and IL-8) possessed great diagnostic accuracy (AUC of 0.86) in distinguishing trauma-induced ALI from controls [14].

The NHLBI ARDS Network study, demonstrated that the combination of clinical risk factors and eight biological plasma markers (VWFAg, SP-D, TNFR1, IL-6, IL-8, ICAM-1, protein C, PAI-1) resulted in a prognostic index for mortality in patients with ALI/ARDS superior to clinical or biological risk factors alone [7]. Model performance was assessed by AUC. AUC increased from 0.815 for clinical predictors and 0.756 for biological markers to 0.85 for the two in combination. A reduced model for mortality that contained APACHE III score, age, SP-D and IL-8 yielded an AUC of 0.834. Data for accuracy, sensitivity and specificity at the optimum discrimination value were not indicated. The great prognostic value of IL-6, IL-8 and SP-D, a surfactant protein and product of alveolar epithelial type II cells for ALI/ARDS outcome demonstrates the significance of alveolar epithelial injury and acute inflammation in the pathogenesis of human ALI/ARDS [7], [12], [38], [39] (this work). Therefore, we examined clinical data and cytokines with CART analysis, since CART analysis is able to handle binary and categorical as well as numeric parameters within one set of data. However, the initially impressive accuracy of a tree including IL-6, IL-8 and Murray's LISS was not stable against cross validation, which demonstrates the need for cross validation for each of the models employed.

In the majority of studies a combination of clinical risk factors and biological markers improved outcome prediction in ALI/ARDS with regard to AUC [7], [14]. In the NHLBI ARDS Network study the additional predictive value of the plasma biomarkers increased AUC moderately from 0.815 for the clinical predictors alone to 0.85 for the combination with plasma biomarkers [7]. As an alternative, we sought to develop a method of outcome prediction which is independent of clinical parameters. This method is based on an acquisition of MALDI-ToF mass spectra of BALF peptides and subsequent mathematical analysis. BALF was chosen because it provides material directly from the alveolar region in the lung where ALI/ARDS takes place [17]. BALF is also a well studied method and is used in the clinical setting of ALI/ARDS for diagnostic purposes in a number of patients. The application of proteomics techniques to BALF has been shown to yield proteins associated with ALI/ARDS [18], [19], [26]–[28]. However, BAL is not without risk. In our study bronchoalveolar lavage was necessary for the diagnosis of bacterial, fungal or viral disease and remaining aliquots were taken for MALDI-ToF MS. Exhaled breath condensate, a possible alternative was also investigated by MALDI-ToF MS [13], [19], [40]. However, the peptide patterns of breath condensate with a limited number of peaks allowed no satisfying outcome prediction.

Patients fulfilling the criteria of ALI/ARDS [41] of the AECC are heterogeneous with respect to the initiating disease entity, age, comorbidity etc. [7]. We were therefore not surprised to learn, that visual inspection of mass spectra did not allow classification of outcome groups (Figure 3). However, mathematical analysis of the spectra did reveal differences in peak patterns. CART analysis predicted outcome with a primary accuracy of 93,7%. However, this number decreased to 76.7% following a 10-fold cross validation. Similar approaches have been reported to identify patients suffering from various lung diseases [18], [25]–[29]. By applying CART analysis to five peptides COPD patients were distinguished from controls with 81.7% accuracy following 10-fold cross validation [29]. The same algorithm used in an analysis of seven predictors in asthma identified these patients with 87.5% accuracy [42]. A CART model of 26 serum proteins (*m/z* and peak intensities) helped to classify healthy controls and patients with lung cancer reaching 90% accuracy [30]. This result however has to be regarded with caution since no cross validation has been applied.

Exclusive peptide biomarkers have not been identified in the BALF of patients with ARDS in contrast to healthy individuals [19]. Instead transient concentration changes of BALF proteins were described at the onset of ARDS. Among those were gelsolin, apolipoprotein A1, the calciumbinding proteins S100A8 and S100A9, complement proteins and antiproteases which all increased whereas surfactant protein-A and fibrinogen were decreased [19], [26]–[28]. This is in agreement with several studies of other respiratory diseases in which peptides exhibited concentration differences in patients when compared to healthy individuals [25], [29], [30].

Recognizing these concentration changes, mathematical algorithms for pattern analysis were applied in order to describe and quantify BALF peptides. SVM algorithm appeared well suited for classification with a limited number of training samples. SVM minimizes training errors and will find a global optimal decision function with maximizing margin which guarantees a minimum test error [43], [44].

Employing SVM based pattern analysis of MALDI-ToF mass spectra in this study resulted in an accuracy of 90% (AUC, 0.953) following 10-fold cross validation with the training group. The quality of this outcome prediction is substantially higher than that based on clinical parameters alone and exceeds that based on clinical parameters plus cytokines (Figure 7, Table 3). Application of this method to a small test group with unknown outcome confirmed the great performance of this test (87.5% accuracy).

SVM has demonstrated its potential in several clinical studies such as the differentiation of phenotypically closely related bacterial species [45], [46]. SVM classifiers were also applied to estimate the prognosis of non-small-cell lung cancer from age, cancer cell type and nine immunomarkers with 76 to 90.5% accuracy [47]. Table 3 summarizes results of analyses with disease markers using SVM algorithms. These results are detailed as accuracies together with area under the curve (AUC) values. AUC represents an accepted measure of the performance of binary classifiers [7], [33]–[35].

One might argue, that performing a MALDI-ToF analysis from bronchial lavage fluid proteins is tedious and expensive. However, once the MALDI-ToF analysis is established, it is very comparable to the determination of cytokines in terms of time and expenses.

This study reveals that the pattern of peptides and proteins in the alveolar lavage fluid by itself includes important information regarding the severity of the disease and the future outcome. Our findings are limited to some extend by the relatively small group size. Apart from larger confirmatory studies faster and more practical techniques might be developed in the future which are based on the combined pattern of mass spectrometry or related methods with clinical data. Another potential benefit might be the identification of patterns and peptides with high prognostic impact and a possible new insight into the pathophysiology of ARDS.

## Materials and Methods

### Patients and Ethics Statement

A total of 46 patients were included in this study. All were mechanically ventilated through an endotracheal tube. Of those 21 did not survive, 25 survived, 28 were male and 18 were female. The mean age was 62±15 years. All patients suffered from severe pneumonia and acute respiratory failure. See Table 4 for ALI/ARDS extent [9], [41] and ventilatory parameters.

**Table 4:** Ventilatory parameters of all patients classified according to both AECC and Murray's LISS definitions.

*Criteria*	*Number of patients*	*PEEP (mbar)*	*PIP (mbar)*	*BF (/min)*	*Tv (ml/kg Bw)*
*Classification of ALI and ARDS of the American-European consensus conference * [*41*]					
Horowitz index ≥300	*n*, 5	7.7±2.1	20.0±4.4	23.0±2.8	6.0±1.1
ALI criteria	*n*, 20	11.2±2.5	23.2±4.4	24.5±5.5	5.6±0.9
ARDS criteria	*n*, 21	11.2±5.7	23.0±5.8	27.4±7.8	5.7±1.2
*Murray's lung injury severity score * [*9*]					
None-to-moderate lung injury (score: 0.1–2.5)	*n*, 27	10.0±3.4	22.0±4.5	24.4±5.7	5.8±1.0
Severe lung injury (score > 2.5)	*n*, 19	12.2±5.2	23.9±5.6	27.4±7.6	5.6±1.2

PEEP indicates positive end-expiratory airway pressure, PIP indicates peak inspiratory airway pressure, BF indicates breathing frequency,

Tv indicates tidal volume adapted to body weight.

Approval (No. 167/2001) for this investigation was received from the ethics committee of the Medical Faculty of the University of Leipzig. Written informed consent was provided by the patients legal representatives. Bronchoalveolar lavage was used in all patients for cytologic, microbiologic and virologic examination on ICU physicians request. For this study an aliquot of the BALF was measured in addition to routine determinations, using flow cytometry and MALDI-ToF MS. All data were processed following anonymization.

### Bronchoalveolar Lavage Protocol

Bronchoalveolar lavage (BAL) was performed in the right middle lobe or lingula between 24 and 96 h following the onset of ventilation. BAL was done according to guidelines [48]. Five 20 ml aliquots of 0.9% NaCl at 21°C were instilled through a fiberbronchoscope and recovered by gentle aspiration [12], [40]. Cells were removed by centrifugation at 450× *g* for 4 min at 8°C. 1 ml aliquots of the supernatant BALF were stored in 1.5 ml Eppendorf tubes at −80°C before use.

### Cytokine Detection in BALF by Flow Cytometry

50 µl BALF were incubated for double determinations with a mixture of six bead populations with distinct fluorescence intensities and coated with capture antibodies specific for IL-1β, IL-6, IL-8, IL-10, IL-12 and TNF-α. We used the cytometric bead array from Becton Dickinson (San Jose, CA, USA). Cytokines were determined by Phycoerythrin(PE)-conjugated detection antibodies with a flow cytometer (FC500, Beckman Coulter). Calibration was performed with standards ranging from 2.5 to 312 pg/ml.

### Evaluation of Clinical Data

The power of clinical features as binary classifiers for outcome was estimated by receiver operating characteristic (ROC) curves. Discrimination power, optimal threshold value, accuracy, sensitivity and specificity were obtained. A *P*-value was calculated which tests the hypothesis that the area under the curve equals 0.5. If *P*<0.05, the corresponding parameter significantly discriminates between survivors and nonsurvivors. A classification and regression tree (CART) analysis identified patients with fatal outcome. Trees were constructed from the training group (n = 30) and applied to both the test group (n = 16) and all patients (n = 46). The tree bagger algorithm of the Matlab statistics toolbox and a random forest algorithm [49] were applied. For calculating ROC curves the training cohort was randomly split into 70% patients in training and the remaining 30% to assess the training performance of the model [50]. This procedure was repeated 50 times to calculate accurately the mean AUC after 10-fold cross validation.

### Peptide Patterns of BALF by Mass Spectrometry

Careful sample preparation is indispensable for the application of matrix-assisted laser-desorption ionization time of flight mass spectrometry (MALDI-ToF MS) to biological materials. Low protein content in BALF, substantial concentrations of surfactant lipids and finally salt from phosphate-buffered saline are all important factors. An overabundance of blood born proteins like albumin and immunoglobulins can also hamper measurements of alveolar lining fluid proteins [15]–[17], [51].

In this study BALF was concentrated approximately 10-fold in a vacuum centrifuge prior to purification by hydrophobic chromatography with a MB-HIC 8-kit (Bruker Daltonics, Germany). Purified peptides were dissolved in 50% acetonitril in 0.1% trifluoroacetic acid and spotted with the matrix α-cyano-4-hydroxy-cinnamic acid (4 mg/ml 50% acetonitril in 0.1% trifluoroacetic acid) on a ground steel target.

Mass spectra were recorded by MALDI-ToF MS from 1,500 to 16,600 mass over charge ratio (*m/z*) using an Autoflex II spectrometer (Bruker Daltonics). 1000 shots were accumulated per spectrum. A mixture of six peptides was utilized for external calibration covering a mass range from 1620.86 to 12360.97 *m/z*. For internal calibration, representative samples were mixed with the peptides and measured again. The raw spectra were processed by baseline subtraction and a slight smoothing with Flex analysis 2.4 (Bruker Daltonics). For peak detection the centroid algorithm with a threshold of signal to noise ratio of 6 was used. For re-calibration of spectra suitable sample peaks were selected as “calibrants”. From each spectrum up to 80 peaks were extracted. Peaks with very low relative intensities (<0.01) were omitted. The peaklists (*m/z* with relative intensity) obtained by this procedure formed the database which was further evaluated by computational analysis.

### Cluster Formation of the Mass Peaks

To refine spectra accuracy all peak lists were aligned for mass drift adjustment [45], [52]. Briefly, a mass-dependent size of the mass window was used according to window size, size_abs_+(size_rel_ * peak mass) with size_abs_, 0.8 *m/z* and size_rel_, 0.001. Thus we arrived at a mean spectrum containing common *m/z* values. All spectra were aligned individually to the peaks of the mean spectrum by linear mass adjustment of the peaks [53]. Subsequently, peak clusters were formed which contained all peaks originating from different individual spectra, however, occurring in the same window. All peaks assigned to one cluster are represented by the respective mean cluster mass. This procedure represents the basis of the mass spectrometric approach.

### Mathematical Analysis of Peptide Pattern of BALF

MALDI-ToF MS data were analyzed by CART and nearest-neighbors classifiers (kNN, k, 1–3) and evaluated by 5-fold cross validation. Finally, classification of the mass spectrometric data and selection of predictive spectral features (candidate peaks) were performed applying a support vector machine (SVM) with a small soft margin parameter. For feature selection, a recursive feature elimination procedure [54], [55] and the shrunken centroid algorithm were used [56]. Matlab 7.8 (The MathWorks, Inc., Natick, MA) including bioinformatics and statistics toolbox was used. Calculations were carried out with the free Spider Matlab machine learning package and the procedure implemented in the Matlab bioinformatic toolbox [54].

## Supporting Information

Figure S1 **Presentation of receiver operating characteristic curves for clinical data and cytokines and spectral features from MALDI-ToF MS.** Receiver operating characteristic (ROC) curves were calculated for classification and regression trees (CART) of both IL-6, IL-8 and Murray's LISS as well as the cluster masses used in Figure 4 (*m/z*, 4468.6, 2719.8, 2052.1 and 2334.9) and support vector machine (SVM) algorithms. The symbols (x, clinical data/cytokines, Δ, spectral features) represent the nodes of the trees. The ROC curve for the SVM algorithm was calculated as described in “Materials and Methods” with an AUC of 0.953 following 10-fold cross validation (CV). The closed circle (•) indicates the point of best classification.
(TIF)

Table S1 Outcome prediction of ALI/ARDS patients based on clinical features/cytokines and spectral features.
(DOC)
