# Supplementary material for: Outcome Prediction in Pneumonia Induced ALI/ARDS by Clinical Features and Peptide Patterns of BALF Determined by Mass Spectrometry
Source: PLoS One. 2011 Oct 3;6(10):e25544. doi: 10.1371/journal.pone.0025544 (PMC3184998; doi:10.1371/journal.pone.0025544)
Supplement: Table S1 — Outcome prediction of ALI/ARDS patients based on clinical features/cytokines and spectral features. (DOC) [file pone.0025544.s002.doc]

**Table S1.** Outcome prediction of ALI/ARDS patients by different predictors.

| *Predictors Algorithm Sensitivity Specificity Accuracy AUC Top peaks (m/z) ∙ (% (%) (%) Rel. importance (%)* | | | | | | | |
| --- | --- | --- | --- | --- | --- | --- | --- |
|  | | | | | | | |
| ***Clinical data / BALF cytokines*** | | | | | | | |
|  | | | | | | | |
| LIS score | ROC | | 73.3 ^1^ | 66.7 ^1^ | 70.0 ^1^ | 0.733^1^ |  |
| IL-6 | ROC | | 82.7 ^1^ | 76.1 ^1^ | 79.4 ^1^ | 0.853^1^ |  |
| IL-8 | ROC | | 80.0 ^1^ | 73.3 ^1^ | 76.7 ^1^ | 0.813^1^ |  |
| IL-6, IL- 8, LISS | CART | | 80.0 ^2^ | 100 ^2^ | 90.0 ^2^ | 0.913^2^ |  |
| IL-6, IL- 8, LISS | CART | | 81.4 | 74.7 | 79.3 | 0.873 |  |
| IL-6, IL- 8, LISS | Rand. forest | | 77.0 | 85.0 | 81.0 |  |  |
| IL-6, IL- 8, LISS | SVM | | 84.0 | 78.0 | 81.0 | 0.840 |  |
|  | | | | | | | |
| ***MALDI-ToF MS, spectral features*** | | | | | | | |
|  | | | | | | | |
| Spectral features (*m/z* and intensity) | | CART | 93.3 ^2^ | 100 ^2^ | 96.7 ^2^ | 0.991^2^ | 4468.6 100, 2719.8 67.3, 2052.1 31.9, 2334.9 31.3 |
| Spectral features | | CART | 67.0 | 67.0 | 70.0 | 0.864 |  |
| Spectral features | | AdaBoost |  |  | 81.8 |  |  |
| Spectral features | | NNBs | 73.3 | 73.3 | 73.3 |  |  |
| Spectral features | | SVM | 93.3 | 86.7 | 90.0 | 0.953 |  |
| (Test group) | | SVM | 83.3 | 90.0 | 87.5 | n.a. |  |

NNBs, nearest-neighbors classifier. n.a., not applicable.

Accuracies and AUC obtained with the training group; values include 10-fold cross validation.

^1^ without cross validation (Simple ROC analysis comprises no cross validation.), ^2^ Cross validation was used to find out the optimal number of nodes.
